# Morphology of lentic and lotic tadpoles from Madagascar

**DOI:** 10.1186/s40850-021-00091-9

**Published:** 2021-09-13

**Authors:** Jörn Laudor, Arne Schulze, Michael Veith, Bruno Viertel, Ortwin Elle, Stefan Lötters

**Affiliations:** 1 Biogeography, Trier University, Universitätsring 15, 54296 Trier, Germany; 2 Hessisches Landesmuseum Darmstadt, Friedensplatz 1, 64283 Darmstadt, Germany; 3 Zoologische Gesellschaft für Arten- und Populationsschutz, Hindenburgstraße 12, 76829 Landau/Pfalz, Germany

**Keywords:** Adaptation, Anura, Larva, Environment, Evolution

## Abstract

**Background:**

The morphology of anuran larvae is suggested to differ between species with tadpoles living in standing (lentic) and running (lotic) waters. To explore which character combinations within the general tadpole morphospace are associated with these habitats, we studied categorical and metric larval data of 123 (one third of which from lotic environments) Madagascan anurans.

**Results:**

Using univariate and multivariate statistics, we found that certain combinations of fin height, body musculature and eye size prevail either in larvae from lentic or lotic environments.

**Conclusion:**

Evidence for adaptation to lotic conditions in larvae of Madagascan anurans is presented. While lentic tadpoles typically show narrow to moderate oral discs, small to medium sized eyes, convex or moderately low fins and non-robust tail muscles, tadpoles from lotic environments typically show moderate to broad oral discs, medium to big sized eyes, low fins and a robust tail muscle.

**Supplementary Information:**

The online version contains supplementary material available at 10.1186/s40850-021-00091-9.

## Background

Amphibians are unique among tetrapod vertebrates by their biphasic and complex life cycle. Most extant amphibians possess free living aquatic larval stages undergoing a drastic bauplan change, called metamorphosis [1, 2]. In the order Anuran, the larval stage is commonly referred to as tadpole. The formation of specialized larval organs for food collection such as the oral disc and the filter apparatus enable tadpoles a broad use of food sources [3] and facilitate the occupation of a variety of niches. It is assumed that this has favoured the extensive anuran radiation [4], with almost 7400 species known today, thus markedly exceeding the number of species in the two other amphibian orders (Gymnophiona, Urodela) with less specialized larvae [5, 6].

Most anurans deposit their eggs in ephemeral or perennial lentic waters [6, 7]. Their larvae have in tendency a globular body, median to high fins, a non-robust tail musculature, a relatively small number of labial tooth rows and a pointed tail end [6]. They are usually allocated to the pond type (here called ‘lentic’). Altig and McDiarmid suggested this type of larvae being close to the hypothetical ancestral larval bauplan [6, 8]. These prevailing morphological traits are frequently found in representatives of most existing phylogenetic lineages [7].

Apart from lentic environments, anuran larvae can use lotic environments for development. Lotic larvae show a much greater morphological diversity than lentic ones [2, 9]. Their morphological features include an enlarged oral disc, a high number of labial tooth rows, an increased number of oral papilla, a depressed body, a robust tail musculature and low fins [6, 10–12]. These characters were altered at different extent and in differing combinations [11–14], and they are interpreted as adaptations to lotic conditions such as the current velocity. This is of particular interest in anuran biology, since the evolutionary change of the larval bauplan is understood as a constraint on the one hand [15], but is highly adaptive on the other hand [2]. Moreover, it is noteworthy, that apparently only a small number of lotic larvae has evolved additional organs in connection with their lotic habitat, such as the ventral sucker in *Atelopus* or *Amolops* tadpoles [6].

In this paper, we study tadpoles from lentic and lotic environments to assess the extent to which morphological alteration of lotic larvae mark a possible response to environmental conditions, namely the water current. A similar study was conducted by Sherratt et al. [16]. The authors used a landmark approach. Landmarks are a precise tool to understand shifts in body shape and body proportions [17]. However, there are limitations when it comes to the analysis of particular morphological features. In anuran larvae, this includes the presence or absence of specialized organs that are known in lotic tadpoles. Likewise, the remarkable morphological variation of tadpoles’ oral structures cannot be assessed using landmarks. Hence, they may perhaps not fully catch the story of the evolution of the anuran larval bauplan to lotic environments in its complexity, why we here suggest a combination of a categorically coded characters and continuous measurements of larvae from lentic and lotic environments.

Madagascan anurans are a suitable ‘model’ group for a comparative study of lotic and lentic tadpoles. First, more than 350 species are known from this island in only five to six radiations (at family and subfamily levels) [5, 18], which provides a high number of candidate species for analysis while, at the same time, reducing the potential impact of phylogenetic constraints on trait evolution. Second, larvae of about one third of all Madagascan species have been described, which is a high number compared to other regions (authors’ unpubl. data). Third, Madagascar is characterized by an enormous landscape diversity [19], offering a wide range of larval habitats.

We expect that our approach will confirm the existence of a general morphospace in which body proportions of larvae can evolve in response to habitat conditions, as shown by Sherratt et al. [16]. We also hypothesize that characters of lotic tadpoles can rather be explained by shared adaptive evolution than by shared synapomorphies.

## Results

### Patterns and univariate analysis

#### Oral disc – position and shape

As shown in Table 1, in lentic tadpoles an anteroventral oral disc position prevails with a normal or folded disc type and a moderate disc width. In lotic larvae, the oral disc position is commonly anteroventral or ventral, while the oral disc type and width are (similar to lentic tadpoles) normal or folded and moderate, respectively. Significant differences (employing a Fisher’s exact rxc-test) in lentic versus lotic tadpoles are found in oral disc position and oral disc width (Table 1). Noteworthy, broad oral discs do only occur in lentic larvae.

**Table 1:** Distribution of character states and results of the Fisher’s exact rxc-tests

	Lentic	Lotic	*P*
Oral disc position			
Ventral	16% (7)	54% (43)	< 0.001*
Anteroventral	67% (29)	36% (29)	
Terminal	16% (7)	10% (8)	
Oral disc type			
Funnel-shaped	0% (0)	9% (7)	0.055
Normal or folded	98% (42)	85% (68)	
Specialised	0% (0)	4% (3)	
No data	2% (1)	3% (2)	
Oral disc width			
Broad	0% (0)	29% (23)	< 0.001*
Moderate	67% (29)	56% (45)	
Narrow	33% (14)	14% (11)	
No data	0% (0)	1% (1)	
Labial tooth rows			
< 5	9% (4)	1% (1)	0.060
5 (includes 2/3)	2% (1)	5% (4)	
> 5	60% (26)	73% (58)	
0 keratodonts	21% (9)	11% (9)	
no data	7% (3)	10% (8)	
Ventral jaw sheath			
V, U, arch	84% (36)	84% (67)	< 0.05*
Arch, V, inverted	0% (0)	6% (5)	
Horizontal	7% (3)	1% (1)	
Specialised	2% (1)	6% (5)	
Missing	5% (2)	0% (0)	
No data	2% (1)	3% (2)	
Dorsal jaw sheath			
V, U, inverted	16% (7)	13% (10)	< 0.05*
Horizontal	2% (1)	0% (0)	
Arch	47% (20)	29% (23)	
Specialised	28% (12)	56% (45)	
Missing	5% (2)	0% (0)	
No data	2% (1)	3% (2)	
Marginal papillae			
1 complete or incomplete row	58% (25)	43% (34)	< 0.05*
≥ 2 complete or incomplete rows	35% (15)	39% (31)	
Specialised	0% (0)	13% (10)	
No papillae	7% (3)	5% (4)	
No data	0% (0)	1% (1)	
Eye position			
More lateral than dorsal	37% (16)	31% (25)	0.832
More dorsal than lateral	12% (5)	14% (11)	
Dorsolateral	51% (22)	55% (44)	
Distance between eyes			
Broad	21% (9)	35% (28)	0.352
Intermediate	51% (22)	54% (43)	
Narrow	12% (5)	8% (6)	
No data	16% (7)	4% (3)	
Eye size			
Big	7% (3)	25% (20)	< 0.01*
Intermediate	74% (32)	71% (57)	
Small	19% (8)	4% (3)	
Body shape			
Depressed	49% (21)	55% (44)	0.187
Moderately depressed	33% (14)	38% (30)	
Normal, globular	19% (8)	8% (6)	
Relative length of tail to body			
< 0, 99	5% (2)	5% (4)	0.988
1-1, 49	14% (6)	15% (12)	
1, 5-1, 99	53% (23)	50% (40)	
≥ 2	28% (12)	30% (24)	
Tail muscle			
Not robust	47% (20)	11% (9)	< 0.001*
Slightly robust	37% (16)	25% (20)	
Robust	16% (7)	64% (51)	
Fins			
Convex shaped, not low	33% (14)	15% (12)	< 0.001*
Moderately low	56% (24)	41% (33)	
Low	12% (5)	44% (35)	
Tail tip			
Pointed	28% (12)	16% (13)	0.299
Intermediate	12% (5)	16% (13)	
Rounded	60% (26)	68% (54)	

Significant values are indicated by *; percentage data are followed by total numbers in parentheses, based on 123 species

#### Oral disc – horny structures and marginal papillae

Most lentic and lotic species have more than five labial tooth rows, one row of marginal papillae and a V- or U-shaped and arched ventral jaw sheath. The dorsal jaw sheath of most lentic species is arched, while most lotic larvae have specialised sheaths (Table 1). In the characters marginal papillae, ventral jaw sheath and dorsal jaw sheath, significant differences between lentic and lotic species are evident (Table 1). Moreover, specialised papillae only occur in lotic larvae and missing jaw sheath only in lentic larvae.

#### Eyes

While commonly lentic and lotic tadpoles have dorsolateral positioned and medium-sized eyes, with a normal eye distance, a significant difference is the relation of eye sizes. Only a few lentic larvae possess big eyes, while most of them have small eyes. In lotic species, this relation is reversed (Table 1).

#### Body and tail

Lentic and lotic tadpoles commonly share a rounded tail tip, which is more than 1.5 times longer than the depressed body. Most lotic larvae have a robust tail musculature and low fins, while lentic ones usually develop moderately low fins and a tail musculature classified as not robust. These differences in tail muscles and fins are highly significant (Table 1).

### Multivariate analysis

A Categorical Principal Component Analysis (CATPCA) reveals that Principal Component (PC) 1 (eigenvalue: 3.557) is exclusively explained by oral structures (Table 2). Species positively correlated with PC1 in 84% of all cases have few (0-1 row) or ‘specialised’, while 46% of those negatively correlated have two or more rows of papillae. The oral disc position of the positively loading species in 48% of all cases is terminal, while those negatively loading are characterized by ventral or anteroventral oral disc positions. Funnel-shaped oral discs are only found in taxa positively correlated with PC1. Finally, most of the species in the negative range of PC1 possess more than five tooth rows (88%), while the majority of the species in the positive range have no or up to five tooth rows (71%).

**Table 2:** CATPCA scores for 15 characters based on tadpoles of 123 anuran species

	Dimension	
	PC1	PC2
IOD/BW	0.383	**0.518**
ED/BL	0.307	**0.521**
ODW/BW	0.080	0.456
TMW/BW	0.080	**0.884**
TMH/BH	−0.053	**0.760**
BH/BW	0.261	0.158
MTH/TMHM	0.317	**−0.593**
Dorsal jaw sheath^a^	−0.309	0.195
Eye position	−0.126	−0.104
Marginal papillae^a^	**0.773**	0.260
Oral disc position^a^	**0.862**	−0.221
Oral disc type	**−0.801**	−0.266
Tail tip	−0.457	0.248
Labial tooth rows	**0.824**	−0.272
Ventral jaw sheath^a^	0.388	−0.302
Eigenvalue	3.557	2.946

Significant factor loadings at ≥│0.5│ are indicated in bold; characters that differ significantly between lentic and lotic larvae in the univariate analysis are marked with ^a^ (cf. Table 1)

The CATPCA shows that regarding PC2 (eigenvalue: 2.946) tail muscle width and height, fin height, eye size and interorbital distance are the decisive parameters with factor loadings ≥│0.5│ (Table 2). PC2 strongly correlates with body and eye characters that show a highly significant difference between lentic and lotic larvae in the univariate analysis. Quantifications of dimensions are provided in the Additional file 1. Tadpoles positively (negatively) correlated with PC2 have in average IOD/BW of 0.64 (0.55), ED/BL 0.15 (0.11), TMW/BW 0.51 (0.37), TMH/BH 0.66 (0.54) and MTH/TMHM of 2.11 (2.71). That is, species positively correlated with PC2 develop bigger and more distant eyes, stronger tail muscles and lower fins than those negatively correlated with PC2.

For detailed data see Additional files 1, 2 and 3.

**Fig. 1:**
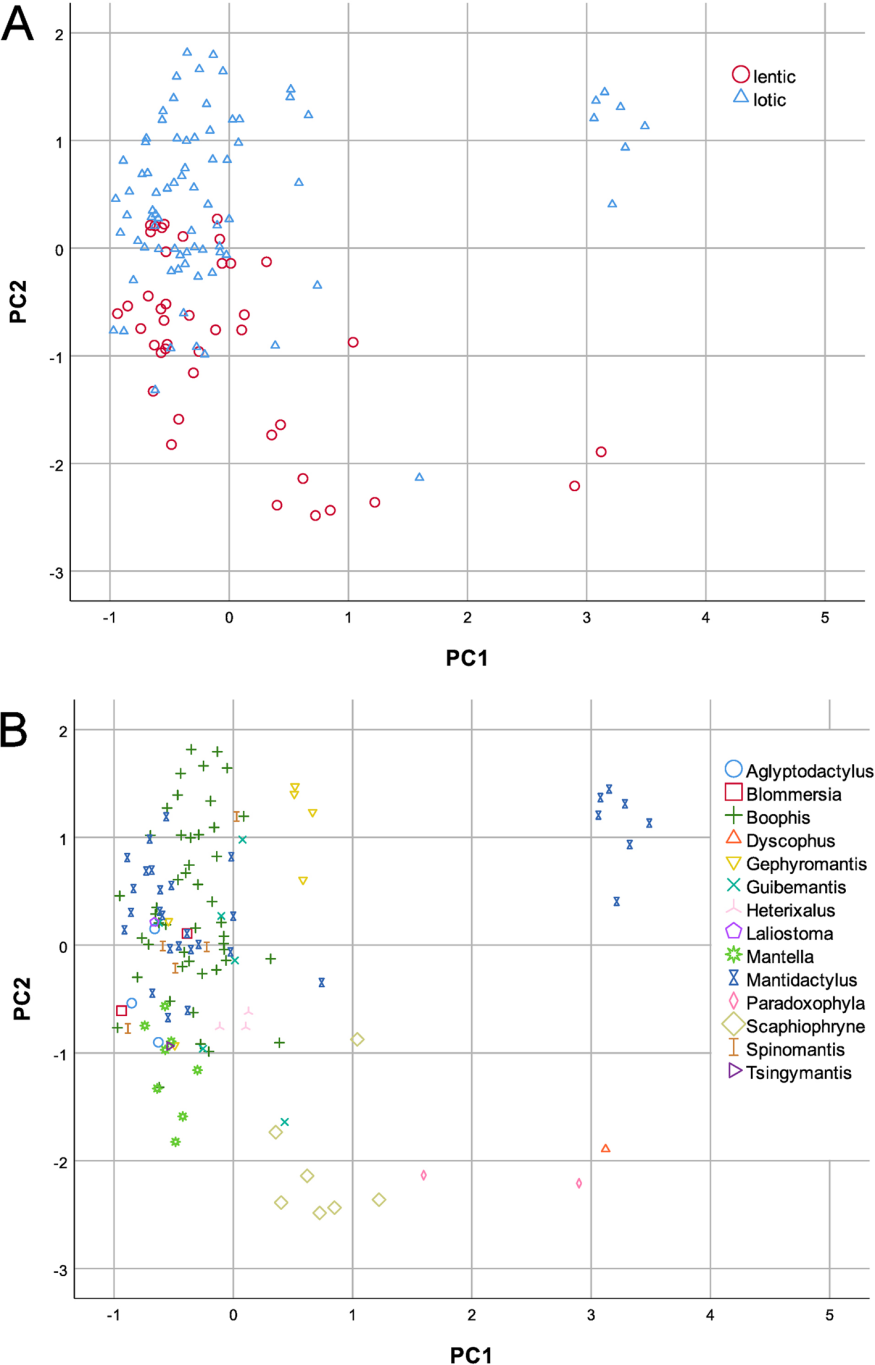
Scatterplots of scores of PC1 and PC2, grouped by (**A**) aquatic habitat (**B**) genus

As visible in Fig. 1a, most tadpoles are grouped at values in the range of − 1 to + 1 of both dimensions. Mainly lotic tadpoles occupy the uppermost range of PC1. They are all members of the subgenus *Chonomantis* of the genus *Mantidactylus* and the (*M. aerumnalis, M. albofrenatus, M. brevipalmatus, M. delormei, M. melanopleura, M. opiparis, M. zipperi*). Congenerics outside this subgenus are scattered elsewhere (Fig. 1b). The only lentic taxa in the uppermost range of PC1 are *Dyscophus insularis* and *Paradoxophyla palmata*; they are clearly distinguished from *Chonomantis* spp. along PC2, however (Fig. 1a, b). A clear gradient along PC2 is obvious from lentic (e.g. genera *Mantella, Scaphiophryne*) to lotic taxa (e.g. *Mantidactylus*, *Boophis*). In-between, larvae from both environments largely overlap at the range − 1 to 0.5. A group of lotic larvae of the genus *Boophis* (*B. albipunctatus, B. andohahela, B. ankaratra, B. luciae, B. mandraka, B. sambirano, B. schuboeae*, *B. vittatus*) is characterized by the uppermost area of PC2 (Fig. 1b), while other members of the genus (both lentic and lotic) are scattered elsewhere. It is noteworthy that, among many lentic tadpoles, only that of *Paradoxophyla tiarano* remains in the lowermost range of PC2 (Fig. 1a, b).

### Phylogenetic effects

With the goal to consider ‘noise’ from underlying synapomorphies in our dataset, we compare the Euclidean distances of PCs and uncorrected p-distances of the 16S rRNA with a Mantel-test. According to this, there is markedly low phylogenetic signal (r^2^ = 0.036, *p* ≤ 0.0001). As the uncorrected p-distance does not account for evolutionary change at the long-term (e.g., reverse or consecutive mutations), we additionally compare the matrix of the best substitution model (GTR + G + I model, see Methods) with that of the Euclidean distances of PCs also using a Mantel-test. Again, this suggests that comparatively little phylogenetic signal occurs (r^2^ = 0.028, *p* ≤ 0.001).

## Discussion

### Characters of lentic versus lotic life

Based on univariate comparisons, we find significant differences between lentic and lotic larvae of Madagascan anuran species for oral disc position, oral disc width, ventral and dorsal jaw sheaths, marginal papillae, eye size, tail muscle and fin shape. Especially, lentic tadpoles always exhibit a narrow to moderate oral disc, almost always small to medium sized eyes and mostly not robust to slightly robust tail muscles with high fins [6, 18, 20]. This is most obvious in *Scaphiophryne* tadpoles [21–23].

The CATPCA also clearly discriminates lentic and lotic tadpoles. An exception is the lotic larva of *Paradoxophyla tiarano*, grouping with the lentic clade. We consider this as a potential bias due to the incomplete data available for the multivariate analysis (cf. Additional file 2). In general and in line to the findings of the rxc-test, tail muscles and fin shape show high factor loadings along PC2 in the CATPCA. As a rule-of-thumb, species positively correlated with PC2 share a lotic habitat and are characterized by bigger eyes, more robust tail muscles and lower fins than the lentic ones which are negatively correlated with the same dimension.

The prevalence of big eyes in lotic tadpoles is difficult to interpret, so their potential functional significance remains to be studied. In contrast, robust tail muscles and low fins have been previously suggested to represent a response to lotic environments [6, 10]. The occurrence of robust tail muscles and low fins in tandem can be interpreted as an adaption to strong currents, because low fins decrease the drag effect and the musculature generates enough thrust to cope with the current [24, 25]. However, robust tail muscles and low fins can be found in lentic tadpoles, as well. They are then found to be associated with surface-feeding [7, 10].

### Additional considerations

In the uppermost range of PC1 (CATPCA; Fig. 1b), all species of *Chonomantis* (subgenus of *Mantidactylus* [26]), and the species *Dyscophus insularis* and *Paradoxophyla palmata* aggregate. The first mentioned have a broad, funnel-shaped and terminally positioned oral disc without any labial tooth rows, combined with an arched dorsal jaw sheath and specialised papillae. These larvae are surface-feeders that exploit the neuston of slow-flowing to stagnant areas in streams, which is possible through a broad funnel-shaped and terminally positioned oral disc [7, 10, 18]. It is obvious that the funnel-shaped oral disc of *Chonomantis* is not an adaptation to lotic life, as similar characteristics occur in lentic larvae such as of the South American hylid (phyllomedusine) genus *Phasmahyla* [20]. Likewise, a terminal oral disc is known from surface-feeding tadpoles in lentic environments [7], so that rather this character is associated with feeding than with lentic vs. lotic environments. Moreover, the arched dorsal jaw sheath does not determinate these tadpoles’ aquatic habitat, as it is a common larval character of the entire subfamily Mantellinae [6, 27, 28], comprising both lentic and lotic species. The specialised papillae of *Chonomantis* tadpoles can also be explained by the particular way of feeding [6, 18]. Likewise, there is evidence that the enigmatic oral disc characteristics of *Dyscophus insularis* and *Paradoxophyla palmata* tadpoles (i.e. absence of marginal papillae, jaw sheaths and labial teeth [19]), are adaptations to microphagous filter-feeding behaviour [29, 30].

Since PC1 is mainly explained by oral structures, we propose that this pattern within PC dimensions does not discriminate lentic versus lotic tadpoles per se. Our results suggest that rather trophic niche specialization may explain this variation along PC1. Additionally, it is necessary to keep in mind that *Chonomantis* is a well-supported monophylum [26]. This study does not provide enough evidence to exclude possible phylogenetic reasons for these tadpoles grouping together along PC1. But in general, as suggested by the result of the Mantel-tests, phylogeny has a minor effect on the arrangement of species along the PCs.

### Methodical considerations

Although with 123 of the more than 350 Madagascan anuran species the proportion of described tadpoles is comparatively high for this region, this list is by far not complete [31]. Even if one considers that there are taxa where free living larvae are missing, e.g. in members of the genus *Gephyromantis* [32, 33], many Madagascan tadpoles obviously remain undescribed, so that we still consider our results as preliminary. Apart from this, the different degrees of accuracy and completeness of the tadpole and habitat descriptions, as well as the ‘subjectivity’ of certain character definitions in Additional file 4 may lead to variation in data quality and a potential bias. This may also apply to the numerous measurements used for the CATPCA that were taken from illustrations of tadpoles (as indicated in Additional file 2).

Moreover, it cannot be ruled out that information obtained from freshly hatched or nearly metamorphosing larvae cause certain ‘noise’. To account for a higher stability within the morphospace, we therefore re-ran the CATPCA for a reduced dataset considering only the 48 species for which data in Gosner [34] stages 30-39 were available (Additional file 2). The results differ slightly in some components, but fins and tail muscles are still suggested to be the most decisive characters delimitating lentic vs. lotic tadpoles. Detailed results are provided in the Additional file 5 (Quantification of categories in Additional file 1).

We suggest that especially the consideration of non-metric characters, such as of the mouthpart, allows for a comprehensive insight of larval aspects related to eco-morphological adaptations. Taking the valuable landmark study by Sherratt et al. [16], only five of our characters would also be mirrored: body shape, relative length of tail to body, fins, tail tip and in parts eye position. Comparing this list to the rxc-test results, only the shape of the fins significantly discriminated between lentic and lotic tadpoles in Madagascan species. On the other hand, in the same test, eight out of 15 additional characters were significantly different between these two tadpole guilds.

In the CATPCA, two out of four landmark-relevant (body shape represented as BH/BW and fins represented as MTH/TMHM) but nine out of fifteen additional characters significantly loaded on the two PCs extracted by us. Hence, aspects of functional morphology, e.g. in the context of adaptation of anuran larvae to lentic or lotic environments, are perhaps best understood in a holistic approach, including landmark data and additional coded morphological characters.

## Conclusion

We show that Madagascan anuran larvae from lentic and lotic environments differ in external morphology and that there is only a limited influence of phylogeny. Lotic tadpoles show moderate to broad oral discs, medium to big sized eyes, low fins and a robust tail muscle. Lentic tadpoles have narrow to moderate oral discs, small to medium sized eyes, convex or moderately low fins and non-robust tail muscles. We thus support the hypothetical existence of a general tadpole morphospace in which body proportions can evolve in response to habitat conditions. It remains to be shown if this patterns (1) still is applicable to the complete dataset of Madagascan tadpoles and (2) if it universally applicable to anuran larvae.

## Methods

Google Scholar and Web of Science were searched for “tadpole” and “Madagascar”, revealing in 31 publications dealing with larval descriptions of 123 Madagascan anurans [18, 20–23, 27, 28, 32, 33, 35–57].

We examined larvae of 43 and 80 species collected in lentic or lotic conditions, respectively. Taxonomy follows Frost [58]; larval stages follow Gosner [34]. All raw data are provided in Additional file 2.

For the Fisher’s exact rxc-test [59], 15 character state conditions were (1) either adopted from published references mentioned in the Additional file 2 or (2) obtained from illustrations therein. We categorized information according to the definitions in Additional file 4: (1) oral disc position: anteroventral, terminal, ventral; (2) oral disc type: funnel-shaped, normal or folded, specialised; (3) oral disc width: broad, moderate, narrow; (4) labial tooth rows: < 5, 2/3, > 5, 0; (5) marginal papillae: 1 complete or incomplete row, ≥ 2 complete or incomplete rows, specialised, no papillae; (6) eye position: more lateral than dorsal, more dorsal than lateral, dorsolateral; (7) distance between eyes: broad, intermediate, narrow; (8) eye size: big, intermediate, small; (9) body shape: depressed, moderately depressed, normal or globular; (10) relative length of tail to body: < 0.99, 1-1.49, 1.5-1.99, ≥ 2; (11) tail muscle: not robust, slightly robust, robust; (12) fins: convex shaped - not low, moderately low, low; (13) tail tip: pointed, intermediate, rounded; (14) ventral jaw sheath: V or U arch, arch or V inverted, horizontal, specialised, missing; (15) dorsal jaw sheath: V or U inverted, horizontal, arch, specialised, missing.

Characters (1), (2), (4)-(6) and (13)-(15) were used as categorical variables in the CATPCA [60]. In addition, morphometric characters were examined (see Abbreviations) follow the definitions of Altig and McDiarmid [6]. Using these characters, seven ratios were computed (1) either using information from references listed in the Additional file 2, or (2) if data not provided, taken with PixelRuler (https://www.pixelruler.de/index.htm, accessed 22 March 2021) from illustrated tadpoles in the respective publications: IOD/BW; ED/BL; ODW/BW; TMW/BW; TMH/BH; BH/BW; MTH/TMHM. We considered variables with factor loadings ≥│0.5│as significantly contributing to the respective PC.

To explore the potential role of phylogenetic signal, we consulted GenBank (https://www.ncbi.nlm.nih.gov/, accessed 22 March 2021) and extracted 16S rRNA sequences of 115 species (for the remaining, no data were available). We used MEGA-X 11 [61] (https://www.megasoftware.net/, accessed 22 March 2021) and the online “robust phylogenetic analysis for everyone” tool [62] (https://ngphylogeny.fr/, accessed 22 March 2021) for the following steps: (1) combining data to a single *fas file (MEGA-X 11), (2) first alignment and cleaning of areas of bad alignments via BMGE and GBLOCKS [62], (3) final alignment and calculating of p-distance matrix (MEGA-X 11), (4) testing for the best substitution model (which is GTR + G + I) of corrected distances and calculating the configuration for the alternative distance matrix using ‘*jmodeltest2*’ [63, 64] (https://github.com/ddarriba/jmodeltest2, accessed 9 July 2021), (5) calculating GTR + G + I model using provided configuration of ‘*jmodeltest2*’ and resulting alternative distance matrix using PAUP* [65] (https://paup.phylosolutions.com, accessed 9 July 2021). Then we calculated the Euclidean distances between PC1 and PC2 of the CATPCA, and finally, employing the ‘*vegan’* [66] package for R, a Mantel-test was performed on both matrices using Spearman-correlation with 9999 permutations.

## Supplementary Information

**Additional file 1.** Quantification of categories.

**Additional file 2.** Data matrix of raw data.

**Additional file 3.** PC scores of examined species.

**Additional file 4.** Definitions of variation of 15 characters, as coded in Additional file 2.

**Additional file 5.** Results of analysis using species in Gosner stages 30-39.

## Data Availability

All data generated or analysed during this study are included in this published article and its additional file.

## Abbreviations

BH: Body height
BL: Body length
BMGE: Block Mapping and Gathering with Entropy
BW: Body width
CATPCA: Categorical Principal Components Analysis
ED: Eye diameter
IOD: Interorbital distance
MTH: Maximum tail height
NCBI: National Center for Biotechnology Information
ODW: Oral disc width
PC: Principal component
TMH: Tail muscle height
TMHM: Tail muscle height at mid-length of tail
TMW: Tail muscle width
