# Supplementary material for: Morphology of lentic and lotic tadpoles from Madagascar
Source: BMC Zool. 2021 Sep 13;6:28. doi: 10.1186/s40850-021-00091-9 (PMC10127085; doi:10.1186/s40850-021-00091-9)
Supplement: Supplementary file 4 — Additional file 4. Definitions of variation of 15 characters, as coded in Additional file 2. [file 40850_2021_91_MOESM4_ESM.docx]

**Definition of variation in 15 characters examined and as coded in Additional file 2.**

**(1)** Oral disc position: *(1)* ventral – in the level of the ventral body wall (V in the illustration below); *(2)* anteroventral – between ventral and terminal (AV); *(3)* terminal – in the extension of the body axis (T).


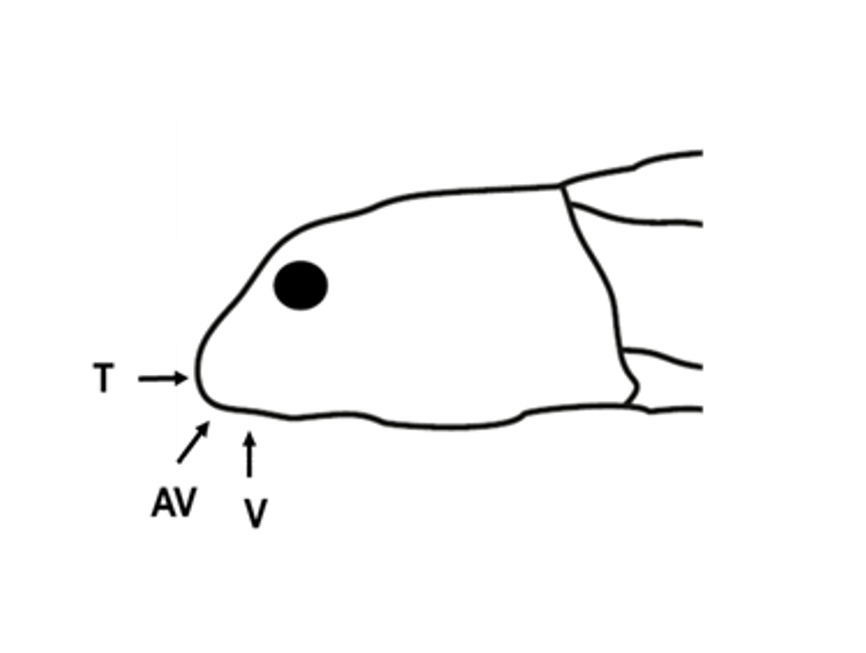


**(2)** Oral disc type: As illustrated below, *(1)* funnel-shaped; *(2)* normal or folded; *(3)* specialised (i.e. not *1* or *2*); *(-1)* no data.


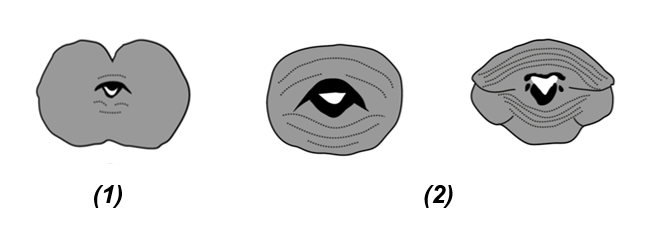


**(3)** Oral disc width: *(1)* broad – if more than entire body width; *(2)* moderate – if between one third and entire body width; *(3)* narrow – if less than one third of the body width; *(-1)* no data.

**(4)** Labial tooth rows *sensu* Altig and McDiarmid (1999): *(1)* < 5 keratodonts; *(2)* 5 keratodonts (including the common LTRF 2/3) and occasionally > 5 keratodonts*; (3)* always > 5 keratodonts; *(99)* 0 keratodonts; *(-1)* no data.

**(5)** Marginal papillae: *(1)* 1 complete or incomplete row; *(2)* ≥ 2 complete or incomplete rows; *(3)* specialised (i.e. not any of the other options); *(99)* no papillae; *(-1)* no data.

**(6)** Eye position: *(1)* more lateral than dorsal; *(2)* more dorsal than lateral; *(3)* dorsolateral.

**(7)** Distance between eyes: *(1)* broad (C in illustration below); *(2)* intermediate (A); *(3)* narrow (B); (-1) no data.

**(8)** Eye size: *(1)* big (C in illustration below), *(2)* intermediate (B, D, E), (3) small (A).


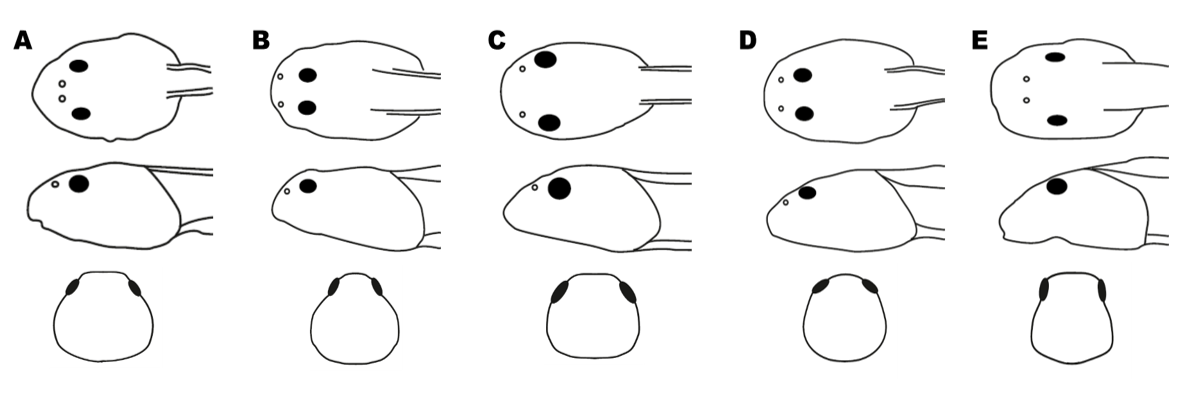


**(9)** Body shape: As illustrated below, from left to right, *(1)* depressed; *(2)* moderately depressed; *(3)* normal or globular.


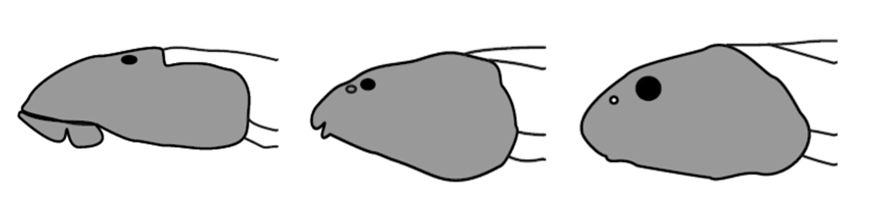


**(10)** Relative length of tail (measured in the centre of the tail musculature at lateral view and from the body) to body: (1) < 0.99; (2) 1-1.49; (3) 1.5-1.99; (4) ≥ 2.

**(11)** Tail muscle: As illustrated below, from left to right, *(1)* not robust; *(2)* slightly robust; *(3)* robust.


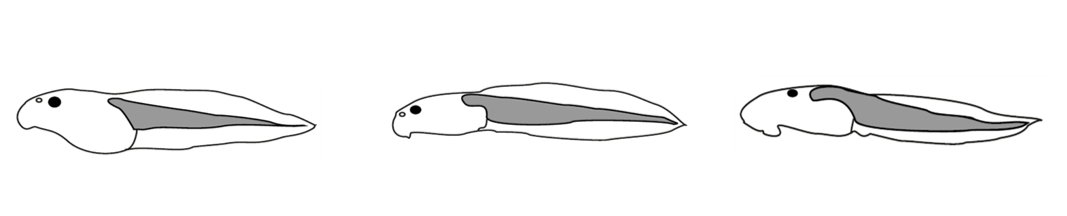


**(12)** Fins: As illustrated below, from left to right, *(1)* convex shaped - not low; *(2)* moderately low; *(3)* low.


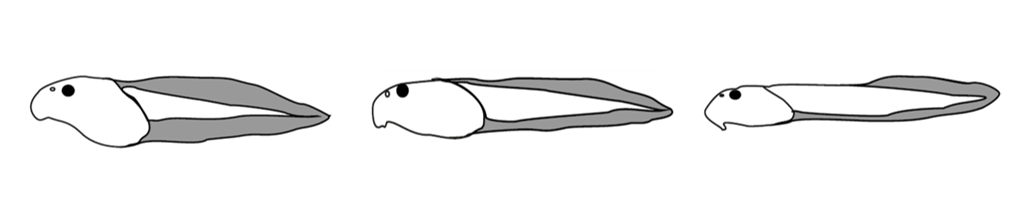


**(13)** Tail tip: As illustrated below, from left to right, *(1)* pointed; *(2)* intermediate; *(3)* rounded.


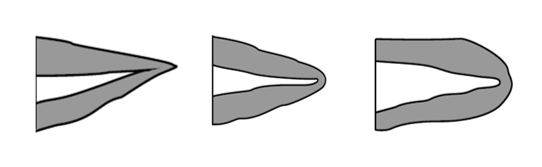


**(14)** Ventral jaw sheath: As illustrated below, from left to right, *(1)* V or U arch; *(2)* arch or V inverted; *(3)* horizontal; *(4)* specialised (i.e. not any of the other options); *(99)* missing; *(-1)* no data.


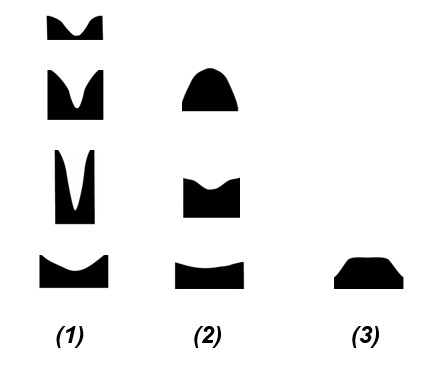


**(15)** Dorsal jaw sheath: As illustrated below, from left to right, *(1)* V or U inverted; *(2)* horizontal; *(3)* arch; *(4)* specialised (i.e. not any of the other options); *(99)* missing; *(-1)* no data.


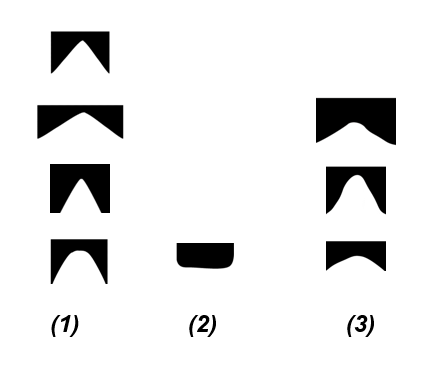


All illustrations by Bruno Viertel.
