# Supplementary material for: Morphology of lentic and lotic tadpoles from Madagascar
Source: BMC Zool. 2021 Sep 13;6:28. doi: 10.1186/s40850-021-00091-9 (PMC10127085; doi:10.1186/s40850-021-00091-9)
Supplement: Supplementary file 5 — Additional file 5. Results of analysis using species in Gosner stages 30-39. [file 40850_2021_91_MOESM5_ESM.docx]

**Results of analysis using species in Gosner stages 30-39.**

To account for a higher stability within the morphospace, a CATPCA with only the 48 species in Gosner stages 30-39 (see Additional file 2) was performed (Table I; Fig. I).

**Table I** CATPCA scores for 15 characters based on tadpoles of 48 anuran species

|  | Dimension | |
| --- | --- | --- |
|  | PC1 | PC2 |
| IOD/BW | 0.103 | **0.703** |
| ED/BL | -0.455 | **0.677** |
| ODW/BW | -0.351 | -0.304 |
| TMW/BW | **-0.808** | 0.415 |
| TMH/BH | **-0.801** | 0.021 |
| BH/BW | 0.281 | 0.492 |
| MTH/TMHM | **0.798** | -0.350 |
| Dorsal jaw sheath | -0.255 | -0.045 |
| Eye position | -0.100 | **-0.670** |
| Marginal papillae | 0.266 | **0.767** |
| Oral disc position | **0.810** | 0.028 |
| Oral disc type | -0.099 | -0.357 |
| Tail tip | -0.493 | 0.090 |
| Labial tooth rows | **0.678** | 0.092 |
| Ventral jaw sheath | **0.512** | **0.516** |
| Eigenvalue | 4.127 | 3.032 |

Significant factor loadings at ≥│0.5│are indicated in bold.


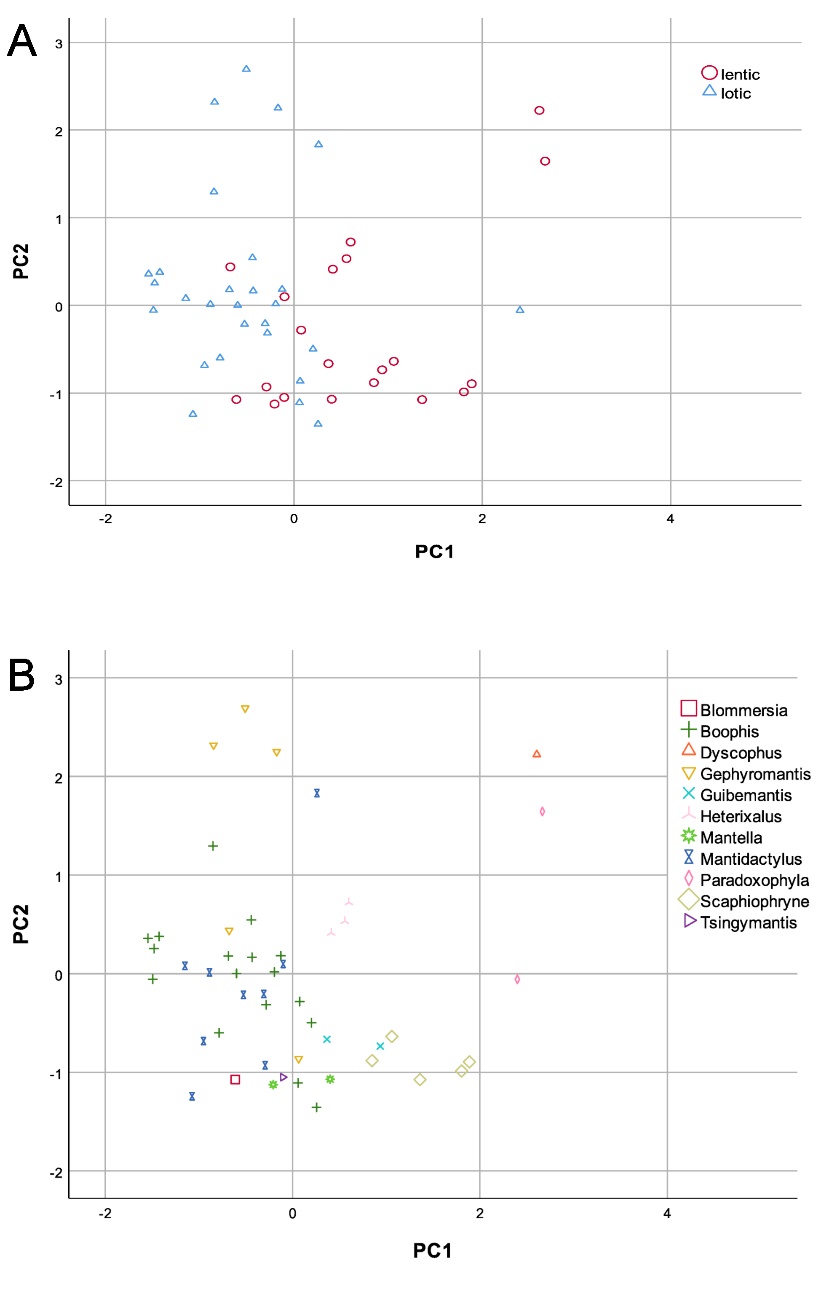


**Figure II** Scatterplots of scores of PC1 and PC2, grouped by (A) aquatic habitat (B) genus

In the analysis considering all 123 species, oral structures were linked to PC1 and habitat was linked to PC2 (Fig.1a, b; main paper). Regarding Figure I, it’s obvious that the dimensions flipped in when analyzing the subset. In PC1, a gradient from solely lotic species in the most negative range to almost solely lentic ones in the positive range is visible. An exception is *Paradoxophyla tiarano*, as discussed in the main paper. The scores for PC1 show that tail muscles and fins are still the decisive values (Table I). The eye distance and relative eye size are now found in PC2, while PC1 is supplemented by oral disc position, tooth rows and ventral jaw sheath.

We suggest that even if the accompanying components differ, the general results of the analysis containing all species is supported – revealing tail muscles and fins are the most important characters distinguishing lentic and lotic tadpoles.

Regarding the phylogenetic impact we also found very low phylogenetic signal: Mantel-test comparing the Euclidean distances of PCs and (1) uncorrected p-distances of the 16S rRNA (r² = 0.054, p ≤ 0.001) and (2) GTR+G+I model distance matrix (r² = 0.0671, p ≤ 0.001).
